# Evaluation of treatment response with serial CT in patients with non-tuberculous mycobacterial pulmonary disease

**DOI:** 10.1007/s00330-024-10987-y

**Published:** 2024-08-01

**Authors:** Sabine Dettmer, Marion Heiß-Neumann, Sabine Wege, Hannah Maske, Felix C. Ringshausen, Oana Joean, Nicole Theissig, Raphael Ewen, Frank Wacker, Jessica Rademacher

**Affiliations:** 1 Institute of Diagnostic and Interventional Radiology, Hannover Medical School, Hannover, Germany; 2 Biomedical Research in Endstage and Obstructive Lung Disease Hannover (BREATH), Member of the German Centre for Lung Research (DZL), Hannover, Germany; 3 Department of Pneumology & Infectious Diseases, Asklepios Lung Clinic Munich-Gauting (CPC-M), Gauting, Germany; 4 Comprehensive Pneumology Center Munich, Member of the German Centre for Lung Research (DZL), Gauting, Germany; 5 Department of Pneumology and Critical Care Medicine, Thoraxklinik at the University Hospital Heidelberg, Heidelberg, Germany; 6 Department of Respiratory Medicine and Infectious Diseases, Hannover Medical School, Hannover, Germany; 7 European Reference Network on Rare and Complex Respiratory Diseases (ERN-LUNG), Frankfurt, Germany

**Keywords:** Nontuberculous mycobacteria, Nontuberculous mycobacterial pulmonary disease, Computed tomography, Treatment response

## Abstract

**Objectives:**

In patients with non-tuberculous mycobacterial pulmonary disease (NTM-PD), the response to treatment is evaluated based on microbiological, clinical, and radiological data. However, little is known about the dynamics of CT findings. The aim of this study was to evaluate CT changes in NTM-PD in order to define radiological criteria for treatment success.

**Methods:**

Retrospective multicenter study (Hannover, Heidelberg, Gauting). Sixty patients with NTM-PD and at least two consecutive CT scans were included. Scoring for NTM-PD was performed by evaluating variables of bronchiectasis, mucus plugging, bronchiolitis, cavities, nodules, and consolidations on an ordinal scale from 0 to 3. Differences between baseline and follow-up were calculated, and patients with/without cultural conversion were compared using the Mann–Whitney *U*-test. For paired comparison of the two consecutive CT scans the Wilcoxon test was used.

**Results:**

Comparing patients with and without culture conversion, there were significant differences in temporal changes of bronchiectasis (*p* < 0.001), cavities (*p* = 0.006), bronchiolitis (*p* < 0.001), consolidations (*p* = 0.004), and total score (*p* < 0.001). Nodules showed no significant differences between groups (*p* = 0.060). The Wilcoxon test showed significant differences between both CTs in patients with a microbiological cure for the total score (*p* < 0.001), cavities (*p* = 0.005), bronchiolitis (*p* < 0.001), and consolidations (*p* = 0.021) with a decrease after microbiological cure, whereas bronchiectasis (*p* = 0.102) and nodules (*p* = 0.18) stayed stable. In the case of persistently positive cultures, there was an increase in the total score (*p* = 0.010) which was attributable to progressive bronchiectasis (*p* < 0.001).

**Conclusion:**

Cavities, consolidations, and bronchiolitis are useful to assess treatment response, whereas bronchiectasis and nodules may remain stable despite successful treatment.

**Clinical relevance statement:**

Cavities, consolidations, and bronchiolitis can assess treatment response whereas bronchiectasis and nodules may remain stable despite successful treatment. In persistently positive cultures, bronchiectasis showed an increase over time indicating that NTM-PD is a progressive chronic disease.

**Key Points:**

- *Little is known about CT changes in nontuberculous mycobacteria pulmonary disease (NTM-PD) and criteria to evaluate treatment response*.
- *In the case of culture conversion, cavities and bronchiolitis decreased whereas bronchiectasis and nodules remained stable*.
- *Cavities and bronchiolitis can evaluate treatment response in NTM, but bronchiectasis and nodules may persist despite successful treatment*.

## Introduction

Nontuberculous mycobacteria (NTM) are ubiquitous environmental bacteria classified in over 190 species and subspecies. Some of them can become human pathogens, while the lung is the most often affected organ (NTM-pulmonary disease (NTM-PD)) [1, 2]. NTM-PD shows a significant increase worldwide. In the USA, the incidence increased from 3.13 per 100,000 in 2008 to 4.73 per 100,000 in 2015 [3]. Moreover, a recent study suggested that a considerable number of NTM-PD cases remain unreported in Germany [4].

Bronchiectasis is the most important underlying chronic lung disease associated with NTM-PD, with a substantial impact on patients’ morbidity and mortality [5, 6]. Chronic airway infection plays a substantial role in its pathogenesis by maintaining a vicious vortex of inflammation and structural damage [7]. The NTM-PD prevalence in patients with bronchiectasis ranges between 1% and 63% [8–10]. In general, it is underestimated, since both the clinical symptoms and the radiological changes are often difficult to distinguish from the underlying lung disease [11].

The diagnosis of NTM-PD is complex and requires clinical symptoms, typical radiological changes, and at least two positive sputum cultures or one culture from bronchoalveolar lavage or from a biopsy [2]. Computed tomography (CT) is the method of choice for radiological evaluation of NTM-PD. Two different forms of pulmonary manifestation are common: the nodular-bronchiectatic form, which is more common in middle-aged women and presents bronchiectasis as structural pulmonary changes, and the fibrocavitary form predominantly in men with previous lung disease [12, 13]. Bronchiectasis, especially in the middle lobes, multifocal bronchiolitis, pulmonary nodules, and cavitation are among the characteristic CT findings of NTM-PD [13–17]. Even though these CT changes are deemed typical for NTM-PD, the sensitivity and specificity of this diagnosis based on CT are low [18]. Bronchiectasis and NTM often occur together and promote each other’s progression; therefore, it is difficult to reliably identify CT morphological signs of active NTM infection in these patients’ lungs.

In patients who meet the clinical, radiological, and microbiological criteria for NTM-PD an antimycobacterial treatment should be considered [2]. Bacteriological success in the treatment of macrolide-susceptible NTM-PD caused by *Mycobacterium avium* species ranges from 71% to 85% [19] with better results in patients treated for more than 12 months [20]. *Mycobacterium abscessus* species is naturally resistant to numerous antimicrobial agents and is responsible for most refractory therapy courses [21]. In addition to the microbiological criteria, the response to NTM-PD therapy is also evaluated based on clinical and radiological data [2]. Chest CT imaging may be beneficial for defining a response to therapy, but there are no precise recommendations for the frequency and interpretation of these evaluations. Given the common occurrence of underlying lung diseases, like bronchiectasis, there is a wide variability in CT findings.

Therefore, we aimed to evaluate CT changes in lung parenchyma after successful therapy of NTM-PD in contrast to patients with cultural NTM persistence in order to define radiological criteria for treatment success.

## Methods

This is a retrospective, multicentre study from Hannover, Heidelberg, and Gauting (Germany). The study was approved by the Internal Review Boards (Hannover: no. 8953_BO_K_2020, Heidelberg: S-619/2023, Gauting: mb23061). We included all patients between the ages of 18 years and 80 years who visited our specialized outpatient services for NTM-PD between January 2015 and February 2024 and who received at least two consecutive CT scans. Patients with severe structural lung changes due to other diseases and patients with cystic fibrosis were excluded.

NTM-PD was diagnosed according to the ATS/IDSA diagnostic criteria [12] with confirmatory microbiologic evidence of NTM infection in the context of clinical and radiographic findings suggestive of NTM-PD. Patients with bronchiectasis received extensive etiological work-up in accordance with the ERS guidelines [22]. Data on clinical factors, including age, sex, body mass index, medical history, and duration of NTM-PD treatment were collected from the electronic medical record system. All sputum-producing patients were examined for mycobacteria at every visit, in accordance with our outpatient services’ routine. In case no spontaneous sputum could be sampled, sputum provocation by hypertonic saline inhalation was considered. Data on culture conversion or NTM persistence was collected from sputum results.

### CT data acquisition

Most CT examinations were obtained at the local Radiology Departments. CT data were acquired volumetrically and reconstructed with a slice thickness of 1.25 mm. Intravenous contrast medium was used if clinically indicated and if there were no contraindications. External CT was accepted if imaging quality was sufficient for evaluation. CT with insufficient quality due to a slice thickness of > 5 mm or severe motion artifacts were excluded. CT examinations were carried out at different time points and for varying clinical questions. For the evaluation, CT examinations were selected at the time of an existing NTM infection (baseline (BL)) and over time, e.g. after therapy (follow-up (FU)). The median interval between the two examinations was 625 days (IQR: 407–965 days).

### CT features and semiquantitative scoring

A radiologist experienced in reading and evaluating chest CT (S.D., 15 years of experience), who was also blinded to the diagnosis and treatment, conducted an evaluation of the CT examinations. All terms were used according to the definition of the Fleischner Society [23]. Bronchiectasis was diagnosed according to the criteria described by Naidich [24].

A scoring for NTM-PD according to Kim et al was performed (Table 1) [25, 26]. Therefore, on an ordinal scale from 0 to 3, bronchiectasis including mucus plugging, cellular bronchiolitis, cavity, nodules, and consolidations were recorded. Regarding bronchiectasis and cellular bronchiolitis, the severity (0 = normal, 1 = less than twice the diameter, 2 = 2–3 times the diameter, and 3 = more than 3 × the diameter of the adjacent pulmonary artery) was registered. Bronchiectasis, mucus plugging, and cellular bronchiolitis, as defined by centrilobular nodules with tree-in-bud pattern (0 = none, 1 = 1–5 segments, 2 = 6–9 segments, and 3 = more than nine pulmonary segments), were registered with regard to their extent. Cavities were scored for the diameter (1 = < 3 cm, 2 = 3–5 cm, and 3 = > 5 cm), wall thickness (1 = < 1 mm, 2 = 1–5 mm, and 3 = > 5 mm) and extent (1 = 1–3 cavities, 2 = 4–5 cavities, and 3 = > 5 cavities). Nodules (1 = 1–5 segments, 2 = 6–9 segments, and 3 = > 9 segments) and consolidations (1 = < 3 segments, 2 = 3–5 segments, and 3 = > 5 segments) were scored according to the number of involved segments [25]. A total score and separate scores for bronchiectasis, cavity, bronchiolitis, nodules, and consolidations were calculated by summing up the individual parameters.

**Table 1:** CT scoring for NTM-PD according to Kim et al [25]

CT finding		Score				SUM score	Total score
		0	1	2	3		
Bronchiectasis	Severity^1^	Absent	Mild	Moderate	Severe	9 Points	30 Points
	Extent^2^	Absent	1–5	6–9	> 9		
	Mucus plugging^2^	Absent	1–5	6–9	> 9		
Cellular bronchiolitis	Severity^3^	Absent	Mild	Moderate	Severe	6 Points	
	Extent^2^	Absent	1–5	6–9	> 9		
Cavity	Diameter (cm)	Absent	< 3	3–5	> 5	9 Points	
	Wall thickness (mm)	Absent	< 1	1–5	> 5		
	Extent^4^	Absent	1–3	4–5	> 5		
Nodules	Extent^2^	Absent	1–5	6–9	> 9	3 Points	
Consolidations	Extent^2^	Absent	1–3	4–5	> 5	3 Points	

^1^ Mild = bronchus diameter greater than adjacent vessel diameter; moderate = bronchus diameter 2–3 times vessel diameter; and severe = bronchus diameter greater than three times vessel diameter

^2^ Data are the number of segments

^3^ Mild = identifiable, peripheral lung,1 cm from pleura; moderate = definite, involvement greater than 1–3 cm from pleura; and severe = extensive, extending to the central lung

^4^ Data are the number of cavities

The sampling date of the first negative culture was the date of culture conversion. Microbiological cure was defined as multiple consecutively negative cultures for the causative species from respiratory samples after culture conversion and until the end of antimycobacterial treatment. The persistence of positive cultures with causative species from respiratory samples while on antimycobacterial treatment, was defined as treatment failure [27].

### Statistical analysis

The IBM SPSS statistics (version 28.0, IBM Corp.) statistical software program was used to analyze the data. Descriptive data were collected and ordinal variables are shown as median with interquartile range (IQR). Differences between BL and FU of each feature and the sum scores were calculated and patients with and without cultural conversion were compared using the Mann–Whitney *U*-test for independent samples. For a paired comparison of the two consecutive CT scans in each patient group, the paired samples Wilcoxon test was used.

## Results

### Patient characteristics

**Fig. 1:**
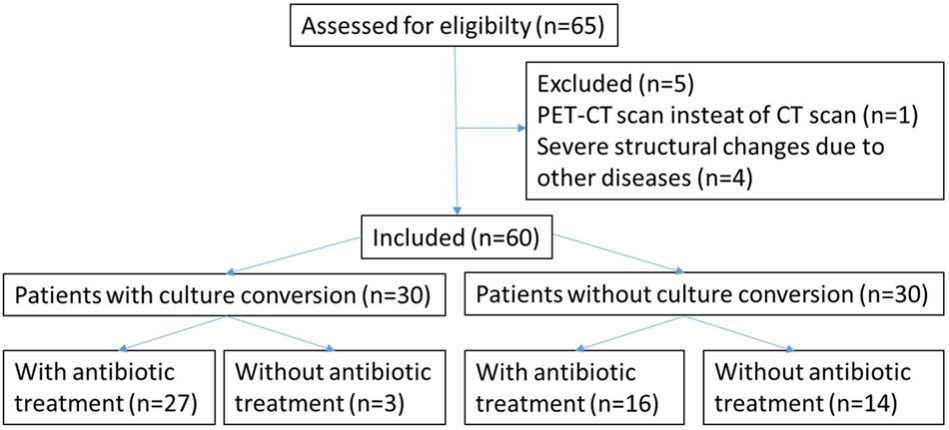
Flow diagram of the study

Sixty-five patients with NTM-PD and two consecutive CT scans could be identified, 40 from Hannover, 10 from Heidelberg, and 15 from Gauting. Five Patients had to be excluded: one patient because of a PET-CT instead of a normal CT scan and four because of severe structural lung changes due to other diseases (alveolar proteinosis, lung carcinoma, allergic bronchopulmonary aspergillosis, and sarcoidosis), resulting in a total number of 60 patients for evaluation (Fig. 1). BL characteristics for patients with NTM-PD are given in Table 2.

**Table 2:** BL characteristics for patients with NTM-PD

Characteristics			NTM-PD, (*n* = 60)
Sex, *n* (%)		Male	16 (27)
		Female	44 (73)
Age, median (IQR)			70 (60–80)
Body mass index, median (IQR)			21 (19–23)
Smoking status, *n* (%)		Never	30 (50)
		Ex	27 (45)
		Active	2 (3)
		Unknown	1 (2)
Pathogen, *n* (%)		*M*. *avium*	34 (57)
		*M*. *intracellulare*	12 (20)
		*M*. *chimaera*	7 (12)
		*M*. *kansasii*	6 (10)
		*M. abscessus* subsp. *abscessus*	4 (7)
		*M*. *abscessus* subsp. *massiliense*	2 (3)
Immunosuppression, *n* (%)			13 (22)
Chronic pulmonary aspergillosis, *n* (%)			7 (12)
Coinfection, *n* (%)	Bacterial coinfection total		26 (43)
	*Pseudomonas aeruginosa*		19 (32)
	*Staphylococcus aureus*		9 (15)
	*Haemophilus influenzae*		2 (3)
	*Achromobacter xylosidans*		1 (2)
	*Stenotrophomonas maltophilia*		1 (2)
	Polymicrobial		4 (7)
NTM-therapy			43 (72)

*NTM-PD* nontuberculous pulmonary mycobacterial disease, *M.* Mycobacterium

Based on the clinical, radiological, and microbiological findings, there was an indication for therapy for all patients. However, only 43 patients received antibiotic treatment. Due to existing comorbidities, the patient’s age, and the patient’s wishes, and after thorough consideration of the advantages and disadvantages of a therapy, 17 patients received no antibiotic treatment. Thirty patients converted, 27 of them with antibiotic treatment and 3 spontaneously. Thirty patients showed no culture conversion, 16 of them with and 14 without antibiotic therapy. Of 43 patients with antibiotic therapy, twenty-six fulfilled the criteria of microbiological cure, while seventeen patients had persistent microbiological evidence for NTM.

### CT scoring for NTM-PD

**Fig. 2:**
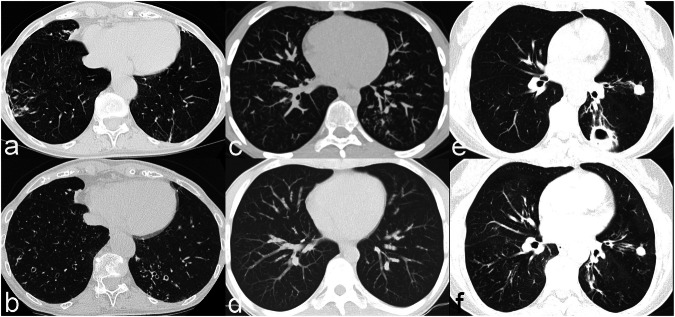
Serial CT in three patients with nontuberculous PD. Patient 1 is a 78-year-old woman with *M. avium* infection. CT shows progressive bronchiectasis between BL (**a**) and FU (**b**) CT 2 years later without antibiotic treatment and persistent cultures. Patient 2 is a 19-year-old male patient with immunosuppression and *M. kansasii* infection. At BL CT (**c**) there were bronchiolitic changes in CT, which disappeared completely in the FU CT (**d**) 1.5 years later after successful antibiotic treatment. Patient 3 is a 60-year-old woman with *M. avium* infection. Cavitary disease in BL-CT (**e**) disappeared in FU CT (**f**) 4 years later after successful antibiotic treatment, whereas nodules persisted

Mann–Whitney *U-*test for independent samples comparing patients with and without culture conversion showed significant differences for temporal changes of the total score for NTM-PD, as well as for the subscores of bronchiectasis, cavities, bronchiolitis, and consolidations (Table 3). Descriptive data reveal that the score and the single features decrease in case of culture conversion and increase in persistent cultures. Nodules are the only characteristic that showed no significant difference between both patient groups. Exemplary CT images are given in Fig. 2.

**Table 3:** Descriptive data and group comparisons with Mann–Whitney *U*-test for patients with and without sputum culture conversion

CT score, (diff FU-BL)	Absent culture conversion, (*N* = 30)	Microbiological cure, (*N* = 30)	*p*-value
	Median, (IQR)	Median, (IQR)	
Total score	1.00 (0.00–2.50)	−3.00 (−5.00 to −0.75)	< 0.001
Bronchiectasis	1.00 (0.00–1.00)	0.00 (0.00 to 0.00)	< 0.001
Cavity	0.00 (0.00–0.00)	0.00 (−1.25 to 0.00)	0.006
Bronchiolitis	0.00 (0.00–0.00)	−1.00 (−3.00 to 0.00)	< 0.001
Nodules	0.00 (0.00–0.00)	0.00 (0.00 to 0.00)	0.060
Consolidations	0.00 (0.00–0.00)	0.00 (−1.00 to 0.00)	0.004

*Diff* difference, *BL* baseline, *FU* follow-up, *IQR* interquartile range

Paired samples Wilcoxon Test with group comparison between BL and FU CT (Table 4) revealed significant differences for the total score (*p* < 0.001) and the sub-scores for cavities (*p* = 0.005), bronchiolitis (*p* < 0.001), and consolidations (*p* = 0.021) for patients with culture conversion with a decrease of all features. Bronchiectasis (*p* = 0.102) and nodules (*p* = 0.180) also decreased slightly but without statistical significance. In case of persistent cultures, the total score (*p* = 0.010) and the sub-score for bronchiectasis (< 0.001) changed significantly over time with an increase between BL and FU. The other CT sub-scores for cavitary disease (*p* = 0.552), bronchiolitis (*p* = 0.796), nodules (*p* = 0.180), and consolidations (*p* = 0.084) were slightly increasing however without statistical significance.

**Table 4:** Descriptive data and group comparisons between BL and FU CT with Wilcoxon test for patients with and without seroconversion

Score	Absent culture conversion, (*N* = 30)			Microbiological cure, (*N* = 30)		
	Baseline	Follow-up	*p*-value	Baseline	Follow-up	*p*-value
	Median, (IQR)	Median, (IQR)		Median, (IQR)	Median, (IQR)	
Total score	11.00 (8.75–14.00)	11.50 (10.00–15.00)	0.010	12.50 (8.75–16.00)	10.00 (4.00–12.25)	< 0.001
Bronchiectasis	4.00 (3.00–6.00)	5.00 (3.75–7.00)	< 0.001	5.00 (3.00–6.25)	5.00 (3.00–6.25)	0.102
Cavity	0.00 (0.00–1.00)	0.00 (0.00–4.00)	0.552	0.00 (0.00–5.25)	0.00 (0.00–0.75)	0.005
Bronchiolitis	4.00 (3.00–5.00)	4.00 (3.00–5.00)	0.796	4.00 (2.00–5.00)	1.00 (0.00–4.00)	< 0.001
Nodules	1.00 (1.00–2.00)	1.00 (1.00–2.00)	0.180	1.00 (1.00–2.00)	1.00 (0.75–2.00)	0.180
Consolidations	0.00 (0.00–1.00)	0.00 (0.00–1.00)	0.084	0.50 (0.00–1.00)	0.00 (0.00–1.00)	0.021

*IQR* interquartile range

## Discussion

In our study, we identified CT changes associated with culture conversion in patients with a diagnosis of NTM-PD. We found that in patients with NTM-PD and microbiological conversion, acute inflammatory changes in CT like cavities, bronchiolitis and consolidations regressed, whereas chronic lung disease (nodules and bronchiectasis) did not show significant improvement. We therefore consider that, in order to assess the response to therapy, primary attention should be paid to bronchiolitic cavitary changes and consolidations whereas persistent nodules and bronchiectasis should not be misinterpreted as treatment failure. In contrast, the total CT score and the sub-score for bronchiectasis increased when cultures remained positive. NTM causes chronically progressive lung disease and triggers the development and progression of bronchiectasis. With persistently positive cultures, long-term deterioration of lung disease must be assumed, whereas bacteriological conversion can delay or even impede the progression of chronic lung diseases such as bronchiectasis.

We evaluated CT for cavities, bronchiolitis, nodules, and bronchiectasis. These patterns were previously described as typical in NTM-PD. Jeong et al identified small nodules, cylindric bronchiectasis, and branching centrilobular nodules as the most common thin-section CT findings of NTM pulmonary infection corresponding histopathologically to bronchiectasis and bronchiolar and peribronchiolar inflammation with or without granuloma formation [17]. Anjos and co-workers analyzed radiological findings derived from 18 articles on NTM-PD with pulmonary cavitation, bronchiectasis, and pulmonary nodules being the most common findings. The middle lobe and lingula show predominant involvement [28]. Further on it is well known, that NTM-PD mostly occurs in patients with underlying chronic lung disease and bronchiectasis and with substantial impact on patients’ morbidity and mortality [5, 6]. It is a vicious circle, in which on the one hand chronic airway inflammation plays a key role in structural damage and development of bronchiectasis, and on the other hand lung disease and in particular bronchiectasis are the most important risk factors for NTM-PD [29, 30]. There is an obvious overlap between CT changes in bronchiectasis and NTM-PD. Therefore, the correct assignment of CT changes and diagnosis of NTM-PD in patients with pre-existing lung disease and bronchiectasis is a diagnostic challenge. Nevertheless, even in patients with bronchiectasis, several patterns like cavitary disease, mucoid impactions, bronchiolitis with the tree-in-bud pattern, pulmonary nodules, and a predominance of bronchiectasis in the middle lobes could be identified as indicative of an additional NTM infection [13, 31].

CT in NTM-PD is used for supporting the diagnosis, determining treatment initiation, and monitoring disease and therapy [2, 27]. A few previous studies have evaluated CT changes as predictors for worsening or recurrent disease in NTM-PD. Structural lung abnormalities are predisposing host factors for NTM infection and recurrence which may be one of the reasons why patients with bronchiectasis have an increased risk of developing NTM-PD [32]. Choi and colleagues identified bronchiectasis, nodules, and consolidation as CT findings that predict the recurrence of disease after successful treatment of NTM-PD [33]. Kitada et al and Lee et al could show that pulmonary cavities and consolidations are both risk factors for disease progression [26, 34].

However, little is known about the dynamics of CT findings during NTM treatment and up to now imaging is not implemented in the definition of treatment success [2, 12, 27]. Lee et al assessed the natural course of the nodular bronchiectatic form of NTM-PD [26]. In a cohort of 265 patients with NTM-PD due to *Mycobacterium avium* complex (MAC), about half of the patients without antibiotic treatment demonstrated progressive disease on serial CT over a mean FU period of 32 months and treatment requirement. Cavities and consolidation on initial CT were associated with a higher risk of progression [26]. In a similar study, Lee et al analyzed serial CT findings in patients with nodular bronchiectatic NTM-PD and antibiotic treatment [35]. In this cohort, lung parenchymal abnormalities showed a significantly decreasing extension on CT after antibiotic treatment, which was mainly related to the improvement of bronchiolitis [35]. This is in agreement with our results showing a progression of CT findings for NTM in persistent cultures and regression after successful treatment and culture conversion. There is also evidence regarding the differences between different subtypes of NTM. In patients with *M. abscessus* subsp. *massiliense*-PD and *M. abscessus* subsp. *abscessus*-PD and antibiotic treatment, Kim et al showed differences in the response to treatment depending on the pathogen [25]. In patients with *M. abscessus* subsp. *massiliense*-PD, culture conversion was achieved in all patients and a decrease in the CT score in the majority (88%) of patients in comparison to a culture conversion rate of 50% and CT score improvement in one-third of patients with *M. abscessus* subsp. *abscessus*-PD. Improvement was notable in cellular bronchiolitis and cavity in *M. massiliense*-PD [25]. Kuroishi et al reported in accordance with our results, that the CT findings of bronchiectasis in patients with NTM-PD did not improve after treatment [36]. Moreover, they showed that nodules and micronodules improved in 70% of cases of MAC-PD after treatment [36]. This is surprising, especially since it is to be assumed that nodules correspond to granulomatous changes. However, since bronchiolitic changes were not evaluated separately in this study, they may have been recorded as micronodules, which could explain the discrepancy in the results. Similarly, Fujiuchi et al found small nodule improvement in CT in patients with NTM-PD and converted sputum culture in a study cohort of 30 patients with MAC-PD which may also represent tree in bud pattern/bronchiolitis changes, as these were not evaluated separately [37].

The decision to initiate antimycobacterial treatment in patients with NTM-PD is often challenging. The most important difficulties include the optimal time point of treatment initiation, weighing the pros and cons of expected success, and drug-related toxicity. Our results and other previous studies indicate that cavities and bronchiolitic changes in CT are reversible and their assessment over time is useful to estimate the success of therapy and to assess response to treatment.

Our study has limitations. It is a retrospective study and selection bias might have been present. However, the recruitment of patients from three different centers should have reduced this bias. We could include 60 patients with NTM-PD, which is a quite small number. The study group was inhomogeneous with different subtypes of NTM, which may have affected radiological outcomes. Nevertheless, the number of patients with NTM-PD is quite high for Western/Central Europe and the differences in the pathogens represent the situation in clinical routine in this patient collective, so we consider the results sufficiently representative.

In conclusion, cavities, bronchiolitis, and consolidations in CT as acute inflammatory changes in NTM-PD decreased in patients with culture conversion, whereas nodules and bronchiectasis indicating chronic lung disease did not show significant improvement. To assess the response to therapy in CT, primary attention should be paid to bronchiolitis, cavitary changes, and consolidations. In contrast, bronchiectasis as chronic lung changes were increasing over time in patients without culture conversion, indicating that NTM-PD is a chronic progressive disease that can be attenuated if successfully treated.

## Abbreviations

BL: Baseline
CT: Computed tomography
FU: Follow-up
IQR: Interquartile range
M: Mycobacterium
MAC: *Mycobacterium avium* complex
NTM: Nontuberculous mycobacteria
NTM-PD: Nontuberculous mycobacterial pulmonary disease
PD: Pulmonary disease
